# Screening for Chronic Obstructive Pulmonary Disease: Validity and Reliability of a Portable Device in Non-Specialized Healthcare Settings

**DOI:** 10.1371/journal.pone.0145571

**Published:** 2016-01-04

**Authors:** Cristina Represas-Represas, Alberto Fernández-Villar, Alberto Ruano-Raviña, Ana Priegue-Carrera, Maribel Botana-Rial, study group of “Validity of COPD-6 in non-specialized healthcare settings”

**Affiliations:** 1 Pulmonology Department, NeumoVigoI+i Research Group, University Hospital Complex of Vigo (CHUVI), Estructura Organizativa de Xestión Integrada de Vigo (EOXI Vigo), BiomedicalResearchInstitute of Vigo (IBIV), Vigo, Spain; 2 Department of Preventive Medicine and PublicHealth, University of Santiago de Compostela, Santiago de Compostela, Spain, CIBER de Epidemiología y Salud Pública, CIBERESP, Madrid, Spain; Lee Kong Chian School of Medicine, SINGAPORE

## Abstract

**Introduction and Objectives:**

The underdiagnosis of chronic obstructive pulmonary disease (COPD) could be improved through screening using portable devices simpler than conventional spirometers in specific healthcare settings to reach a higher percentage of the at-risk population. This study was designed to assess the validity and reliability of the COPD-6 portable device to screen for COPD in non-specialized healthcare settings.

**Methods:**

Prospective cohort study to validate a diagnostic test. Three cohorts were recruited: primary care (PC), emergency services (ES) and community pharmacies (CPh). Study population: individuals with risk factors for COPD (>40 years, smoking >10 pack-years, with respiratory symptoms). The values measured using the COPD-6 were FEV1, FEV6 and the FEV1/FEV6 ratio. Subsequently, participants underwent conventional spirometry at hospital, using a post-bronchodilator FEV1/FVC value <0.7 as the gold standard criterion for the COPD diagnosis.

**Results:**

437 participants were included, 362 were valid for the analysis. COPD was diagnosed in 114 patients (31.5%). The area under the ROC curve for the COPD-6 for COPD screening was 0.8.The best cut-off point for the FEV1/FEV6 ratio was 0.8 (sensitivity, 92.1%) using spirometry with the bronchodilator test as the gold standard. There were practically no differences in the COPD-6 performancein the different settings and also regarding age, gender and smoking status.

**Conclusions:**

The COPD-6 device is a valid tool for COPD screening in non-specialized healthcare settings. In this context, the best cut-off point for the FEV1/FEV6 ratio is 0.8.

## Introduction

Chronic obstructive pulmonary disease (COPD) is a very common disease, with a prevalence of 10.2% in Spain, higher in men than in women, and its frequency increases with age. However, underdiagnosis can be as high as 73%[1,2]. The ability to perform spirometry—essential test for COPD diagnosis—in primary care (PC) centres would improve this situation, and such a recommendation has been made by the Spanish Society of Pneumology and Thoracic Surgery (SEPAR)[1] and by the National COPD Strategy of the Spanish National Health System[2]. However, in PC we find not only a limited availability of spirometry but also that the test is inadequately performed in a large number of centres[3–5], though this varies considerably between the different Spanish regions[6,7].

One of the causes of an insufficient quality of spirometry is difficulty in obtaining the forced vital capacity (FVC) correctly. An alternative parameter, the forced expiratory volume in six seconds (FEV6), has therefore been accepted as a substitute[8,9]. This simplifies spirometry technique and thus improves accuracy in the diagnosis of airway obstruction in non-specialist settings[6]. In addition, if our aim is to improve out-of-hospital screening for COPD to reduce underdiagnosis, a key element would be to employ portable devices that are easier to operate than conventional spirometers, such as the Vitalograph COPD-6 device[10].

In view of the recognised benefits of the early diagnosis of COPD, both the Spanish COPD Strategy[2] and GesEPOC (the Spanish COPD Guidelines)[1] recommend proactive screening in individuals at-risk. PC would appear to be ideal for the implementation of these programs[1,2,11], but testing could also be extended to those settings in which there are health professionals with basic training in performing respiratory function tests, and easily accessible to the general public, such as community pharmacies (CPh), where many users report respiratory symptoms (without having consulted their doctor), and emergency services (ES), where patients may attend for an exacerbation. Spirometry with a bronchodilator test, the gold standard, should then be used to confirm a suspected diagnosis.

The COPD-6 device measures FEV1 and FEV6 and calculates its ratio. Our group has already validated this device for the detection of obstructive respiratory disease in a hospital setting[12]. The next step would be to demonstrate whether the COPD-6 device could also be useful as a screening tool for COPD in the out-of-hospital setting, administered by staff who does not have extensive experience in performing spirometries.

The objective of this study was to establish the validity and reliability of the COPD-6 device to screen for COPD among individuals at high COPD risk in non-specialized healthcare settings (PC, ES and CPh). Conventional spirometry with a bronchodilator test was used as the gold standard for comparison. This objective falls within the scope of COPD research proposed in the official declaration of the American Thoracic Society (ATS) and the European Respiratory Society (ERS)[13].

## Material and Methods

Prospective, multi-cohort study to validate a diagnostic test in various settings. The study was carried out between 2011 and 2014 in the Vigo University Hospital Complex (CHUVI) in Galicia, Spain, using consecutive cohorts: 1) a cohort drawn from PC patients (PC cohort), with the participation of 8 Health Centres in the Vigo Health District in Galicia, Spain; 2) a cohort recruited from users of Community Pharmacies(CPh cohort), with the participation of 15 pharmacies in the Vigo area, with the collaboration of the Pontevedra Official College of Pharmacists; and 3) a cohort recruited from patients attending the emergency services (ES cohort), undertaken in of the CHUVI (Xeral Hospital and Meixoeiro Hospital) and in the Redondela and Porriño emergency services for PC.

Ten PC doctors, 13 hospital and out-of-hospital ES doctors and 15 pharmacists participated in the study. All were previously trained in the use of the COPD-6 device in a 2-hour theoretical and practical session given by chest physicians and nurses who were participating in the study.Training timerequired to perform conventional spirometries adequately is longer.

### Study population

Individuals who attended a PC consultation (for a respiratory or other problem), who used a participating CPh or who presented to the hospital or out-of-hospital ES with symptoms of a respiratory exacerbation, were asked to participate in the study if they satisfied the inclusion criteria.

#### Inclusion criteria

The same inclusion criteria were used for all 3 cohorts: patients aged over 40 years, smokers or ex-smokers of more than 10 pack-years, and symptoms suggestive of COPD (dyspnea, cough, sputum production). These criteria were consistent with the recommendations of the Spanish COPD Strategy[2] and GesEPOC[1], though it should be recognised that the GesEPOC recommendations were published after initiation of the first phase of our study and lowered the age criterion for the high-risk group to 35 years.

#### Exclusion criteria

The following exclusion criteria were applied: the individual declined to participate in the study, was unable to attend the hospital to perform the test for diagnostic confirmation, or presented any of the absolute contraindications for performing spirometry[14]. Individuals who had already been diagnosed with a respiratory disease were also excluded.

Study participants were recruited consecutively in a non-systematic manner after the participating doctor or pharmacist (in PC, ES or CPh) had checked that they satisfied the inclusion criteria and had obtained written informed consent after explaining the nature of the study.

The studies of each cohort were approved by the Galician Ethics Committee (dossier number: 2010–73, 2011–284, 2012–389).

### Procedures

Measurement of the functional parameters, FEV1, FEV6 and FEV1/FEV6 ratio, were performed using the portable COPD-6 device (model 4000, Vitalograph Ltd., Ennis, Co. Clare, Ireland). The patient was required to perform manoeuvres similar to those used in forced spirometry with the difference that, in this case, it was only necessary to maintain exhalation for 6 seconds. Measurements were repeated until at least 2 reliable measurements were achieved, and the best values obtained for each patient were recorded. The participating PC physicians and pharmacists (for the PC and CPh cohorts) then contacted the chest physicians of the research team by mobile phone to schedule the second phase of the study, which was carried out within the following 24 hours. In the case of the ES cohort, the participating doctors in the ES remitted the patients’ data to the research team by internal post; as the patients in this cohort had attended because of an exacerbation, they were scheduled for the hospital phase of the study one month after the emergency consultation, during a clinically stable phase.

In the hospital, blinded to the results of the tests performed with the COPD-6 device, the research staff performed conventional spirometry using a Datospir 120 spirometer (Sibelmed, Barcelona, Spain), recording the FEV1, FVC and FEV1/FVC ratio. The test was performed in accordance with SEPAR guidelines[14]. When an FEV1/FVC ratio less than 0.7 was detected, the study was completed with a bronchodilator test, with measurement of the same parameters after the administration of 400 μg of salbutamol via a spacer device.

### Statistical analysis

A descriptive analysis of the variables was performed for each cohort and for the overall sample. Sensitivity (S), specificity (Sp), positive predictive value (PPV), negative predictive value (NPV) and the positive and negative likelihood ratios (PLR/NLR) were calculated for the different cut-off points of the FEV1/FEV6 ratio.

The area under the receiver operating characteristic (ROC) curve was determined for the FEV1/FEV6 ratio calculated using the COPD-6 device employed to screen for COPD. An FEV1/FVC ratio less than 0.7 obtained on spirometry after the bronchodilator test was used as the gold standard criterion for the diagnosis of COPD. In addition, the areas under the ROC curves adjusted for a series of variables were compared using the Chi-squared test for homogeneity of areas, taking the median value as the cut-off point for age and for pack-years of the cumulative smoking history.

The analysis was performed using the Statistical Package for Social Sciences version 15.0 (SPSS, Chicago, Illinois, USA).

Presentation of the study was based on STARD methodology for reporting studies of diagnostic accuracy.

## Results

A total of 437 individuals were included in the three cohorts; 362 subjects were valid for analysis (75 were withdrawn because they presented exclusion criteria or did not attend the hospital visit).

The characteristics of the participants in each of the cohorts and in the overall sample are shown in Table 1. The mean age of the participants in the overall study population was 55 years, 61.9% men, and 72.4% active smokers.

COPD was diagnosed in 114 patients, a prevalence of 31.5%; the majority (96 patients, 84.2%) were in Global Initiative for Chronic Obstructive Lung Disease (GOLD) stage I or II.

**Table 1:** Characteristics of the patients in the three cohorts and in the overall sample.

	PC Cohort	CPh Cohort	ES Cohort	Overall Sample
Participants	167	143	127	437
Excluded/ Lost to follow-up	17	33	25	75
Valid for analysis	150	110	102	362
Age, years, mean (SD)	56.8 (9.7)	53.1 (8.6)	56 (11.1)	55.4 (9.9)
Men, n (%)	106 (70.6%)	44 (40%)	73 (71.6%)	224 (61.9%)
Active smokers	112 (74.6%)	85 (77.3%)	65 (63.7%)	262 (72.4%)
Cumulative tobacco consumption in pack-years, mean (SD)	39 (21.6)	32.5 (17)	31.8 (19.1)	35 (19.8)
COPD	60 (40%)	22 (20%)	32 (31%)	114 (31,5%)
GOLD stage I	12 (20%)	8 (36.4%)	13(40.6%)	33 (29%)
GOLD stage II	37 (61.7%)	11 (50%)	15 (46.9%)	63 (55.2%)
GOLD stage III	10 (16.7%)	3 (13.6%)	4 (12.5%)	17 (15%)
GOLD stage IV	1 (1.7%)	0	0	1 (0.8%)

Abbreviations: COPD, chronic obstructive pulmonary disease; CPh, community pharmacies; ES, emergency services; GOLD, Global Initiative for Chronic Obstructive Lung Disease; PC, primary care.

**Fig 1:**
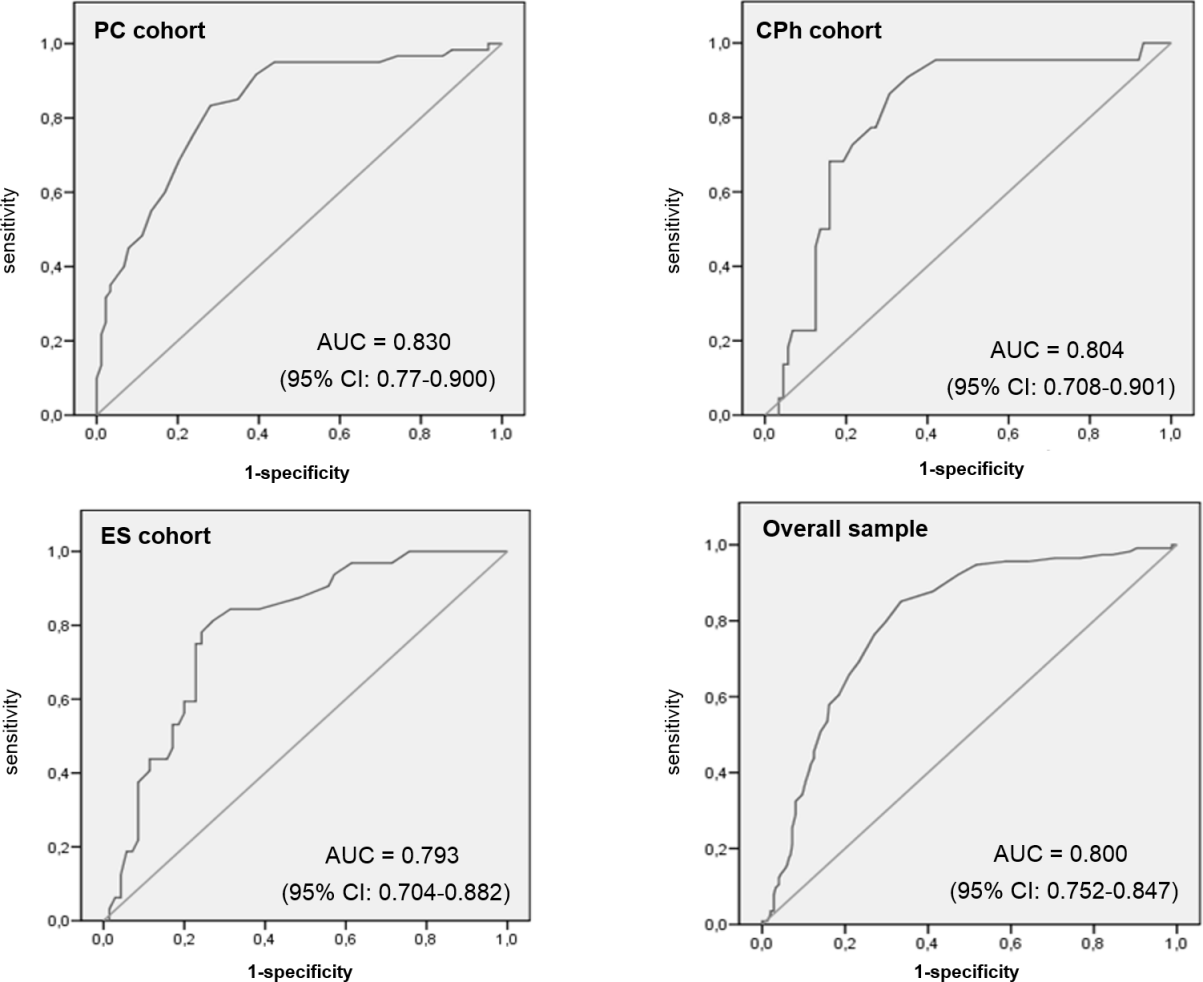
ROC curves of the FEV1/FEV6 ratio measured using the COPD-6 device to screen for chronic obstructive pulmonary disease in primary care (top left), community pharmacies (top right), emergency services (bottom left) and in the overall sample (bottom right). Abbreviations: AUC, area under curve; CI, confidence interval; CPh, community pharmacies; ES, emergency services; GOLD, Global Initiative for Chronic Obstructive Lung Disease; PC, primary care; ROC, receiver operating characteristic.

The ROC curves of the FEV1/FEV6 ratio calculated by the COPD-6 are shown in Fig 1 for each of the cohorts and for the overall patient sample, in which the area under the curve (AUC) was 0.8 (95% confidence interval, 0.752–0.847).

A comparison was also performed of the area under the COPD-6 ROC curves as a function of sex, age, smoker status and cumulative smoking history; no significant differences were found between these groups. These results are shown in Table 2. We also observed that COPD 6 is valid for screening even in more severe categories of COPD. With the cutoff point of 0.8, no patient with severe COPD was false negative in the screening.

**Table 2:** Comparison of the area under the ROC curves of the FEV1/FEV6 ratio measured by the COPD-6 device, according to sex, age and smoking history.

		Area Under the ROC Curve	*P*
Sex	Male	0.803 (95% CI, 0.744–0.862)	0.99
	Female	0.803 (95% CI, 0.721–0.885)	
Age	< 55 y	0.816 (95% CI, 0.746–0.886)	0.48
	≥ 55 y	0.782 (95% CI, 0.714–0.849)	
Smoking	Active smoker	0.837 (95% CI, 0.776–0.897)	0.27
	Ex-smoker	0.785 (95% CI, 0.713–0.856)	
Cumulative tobacco consumption	< 30 pack-years	0.822 (95% CI, 0.754–0.889)	0.34
	≥ 30 pack-years	0.777 (95% CI, 0.711–0.842)	

Abbreviation: CI, confidence interval.

Tables 3–6 show the sensitivity (S), specificity (Sp), positive predictive value (PPV), negative predictive value (NPV),positive likelihood ratio (PLR) and negative likelihood ratio (NLR)for the different cut-off points of the FEV1/FEV6 ratio in the PC, CPh and ES cohorts and in the overall study population. Based on the overall population, a cut-off point of 0.8 for the FEV1/FEV6 ratio had a S of 92.1%, Sp of 52.8%, NPV of 93.6% and PPV of 47.3% for the detection of COPD.

**Table 3:** Primary Care cohort. Values for sensitivity, specificity, positive predictive value, negative predictive value, positive likelihood ratio and negative likelihood ratio for different cut-off points for the FEV1/FEV6 ratio determined during screening for COPD using the COPD-6 device in PC (gold standard criterion for comparison: post-bronchodilator spirometry FEV1/FVC <0.7).

FEV1/FEV6 (COPD-6)	S	Sp	PPV	NPV	PLR	NLR
**0.70**	38.3%	93.3%	79.3%	69.4%	5.75	0.66
**0.71**	40%	92.2%	77.4%	69.7%	5.14	0.65
**0.72**	45%	91.1%	77.1%	71.3%	5.06	0.6
**0.73**	48.3%	87.8%	72.5%	71.8%	3.95	0.59
**0.74**	55%	85.6%	71.7%	74%	3.81	0.53
**0.75**	60%	82.2%	69.2%	75.5%	3.38	0.49
**0.76**	68.3%	78.9%	68.3%	78.9%	3.24	0.4
**0.77**	75%	75.6%	67.2%	81.9%	3.07	0.33
**0.78**	83.3%	71.1%	65.8%	86.5%	2.88	0.23
**0.79**	85%	64.4%	61.4%	86.6%	2.39	0.23
**0.80**	91.7%	60%	60.4%	91.5%	2.29	0.14
**0.81**	95%	55.6%	58.8%	94.3%	2.14	0.09

Abbreviations: NLR, negative likelihood ratio; NPV, negative predictive value; PLR, positive likelihood ratio; PPV, positive predictive value; S, sensitivity; Sp, specificity.

**Table 4:** Community pharmacies cohort. Values for sensitivity, specificity, positive predictive value, negative predictive value, positive likelihood ratio and negative likelihood ratio for different cut-off points for the FEV1/FEV6 ratio determined during screening for COPD using the COPD-6 device in CPh (gold standard criterion for comparison: post-bronchodilator spirometry FEV1/FVC <0.7).

FEV1/FEV6 (COPD-6)	S	Sp	PPV	NPV	PLR	NLR
**0.70**	68.2%	84.1%	51.7%	91.4%	4.29	0.38
**0.71**	68.2%	80.7%	46.9%	91.0%	3.53	0.39
**0.72**	68.2%	80.7%	46.9%	91.0%	3.53	0.39
**0.73**	72.7%	78.4%	45.7%	92.0%	3.37	0.35
**0.74**	77.3%	73.9%	42.5%	92.9%	2.96	0.31
**0.75**	77.3%	72.7%	41.5%	92.8%	2.83	0.31
**0.76**	86.4%	69.3%	41.3%	95.3%	2.81	0.20
**0.77**	86.4%	69.3%	41.3%	95.3%	2.81	0.20
**0.78**	90.9%	64.8%	39.2%	96.6%	2.58	0.14
**0.79**	95.5%	58%	36.2%	98.1%	2.27	0.08
**0.80**	95.5%	51.1%	32.8%	97.8%	1.95	0.09
**0.81**	95.5%	44.3%	30%	97.5%	1.71	0.10

Abbreviations: NLR, negative likelihood ratio; NPV, negative predictive value; PLR, positive likelihood ratio; PPV, positive predictive value; S, sensitivity; Sp, specificity.

**Table 5:** Emergency services cohort. Values for sensitivity, specificity, positive predictive value, negative predictive value, positive likelihood ratio and negative likelihood ratio for the different cut-off points for the FEV1/FEV6 ratio determined during screening for COPD using the COPD-6 device in ES (gold standard criterion for comparison: post-bronchodilator spirometry FEV1/FVC <0.7).

FEV1/FEV6 (COPD-6)	S	Sp	PPV	NPV	PLR	NLR
**0.70**	62.5%	77.1%	55.6%	81.8%	2.73	0.49
**0.71**	68.8%	77.1%	57.9%	84.4%	3.0	0.40
**0.72**	75%	77.1%	60%	87.1%	3.28	0.32
**0.73**	75%	75.7%	58.5%	86.9%	3.09	0.33
**0.74**	78.1%	75.7%	59.5%	88.3%	3.21	0.29
**0.75**	81.3%	72.9%	57.8%	89.5%	3.0	0.26
**0.76**	84.4%	68.6%	55.1%	90.6%	2.69	0.23
**0.77**	84.4%	62.9%	50.9%	89.8%	2.27	0.25
**0.78**	84.4%	61.4%	50%	89.6%	2.19	0.25
**0.79**	87.5%	51.4%	45.2%	90%	1.80	0.24
**0.80**	90.6%	44.3%	42.6%	91.2%	1.63	0.21
**0.81**	93.8%	42.9%	42.9%	93.8%	1.64	0.14

Abbreviations: NLR, negative likelihood ratio; NPV, negative predictive value; PLR, positive likelihood ratio; PPV, positive predictive value; S, sensitivity; Sp, specificity.

**Table 6:** Overall sample. Values for sensitivity, specificity, positive predictive value, negative predictive value, positive likelihood ratio and negative likelihood ratio for the different cut-off points for the FEV-1/FEV6 ratio determined during screening for COPD using the COPD-6 device in non-specialist healthcare settings (gold standard criterion for comparison: post-bronchodilator spirometry FEV1/FVC <0.7).

FEV1/FEV6(COPD-6)	S	Sp	PPV	NPV	PLR	NLR
**0.70**	50.9%	85.9%	62.4%	79.2%	3.61	0.57
**0.71**	53.5%	84.3%	61%	79.8%	3.41	0.55
**0.72**	57.9%	83.9%	62.3%	81.3%	3.6	0.5
**0.73**	60.5%	81.5%	60%	81.8%	3.27	0.48
**0.74**	65.8%	79%	59.1%	83.4%	3.13	0.43
**0.75**	69.3%	76.6%	57.7%	84.4%	2.96	0.4
**0.76**	76.3%	73%	56.5%	87%	2.83	0.32
**0.77**	79.8%	70.2%	55.2%	88.3%	2.68	0.29
**0.78**	85.1%	66.5%	53.9%	90.7%	2.54	0.22
**0.79**	87.7%	58.9%	49.5%	91.3%	2.13	0.21
**0.80**	92.1%	52.8%	47.3%	93.6%	1.95	0.15
**0.81**	94.7%	48.4%	45.8%	95.2%	1.84	0.11

Abbreviations: NLR, negative likelihood ratio; NPV, negative predictive value; PLR, positive likelihood ratio; PPV, positive predictive value; S, sensitivity; Sp, specificity.

## Discussion

This study is the first to show that the COPD-6 is a valid and reliable tool for the detection of COPD in different non-specialized healthcare settings. The validity indexes obtained are very good and show no significant differences according to sex, smoking history or age. Furthermore, the results hardly differ when the COPD-6 is applied in different settings and for different conditions such as gender, age group or smoking status. These results reinforce the validity and reliability of the COPD-6 test.

COPD screening is important because the early detection of COPD and intervention for smoking cessation can delay lung function decline, reduce the burden of COPD symptoms, reduce healthcare costs, and improve patient´s quality of life[2]. In a previous study in the hospital setting, our group has validated the COPD-6 for the detection of obstructive pulmonary disease[12]. The results of the present study are highly relevant because they provide evidence of the reliability of simple spirometric manoeuvres with an easy-to-use device to COPD screening, performed by staff who have not been trained in pulmonary function testing and who have only received a brief theoretical-practical course on the use of the device.

The use of the FEV1/FEV6 ratio as a substitute for the FEV1/FVC ratio has already been accepted[8,9].According to the PLATINO longitudinal study data, FEV1/FEV6 is a better indicator of airflow obstruction, likely due to comparing volumes at fixed times of the expiratory manoeuvre and avoiding inconsistencies due to changes in the quality of the spirometries, especially in forced expiratory time across different technicians, centres, or along time[15]. Postbronchodilator FEV6 and FEV1/FEV6 as percentage of predicted are also independent prognostic factors in stable outpatients with COPD[16]. In any case,there is no consensus on the FEV1/FEV6 cut-off point considered to define obstruction[8,9,12]. The election of the cut-off point for the FEV1/FEV6 ratio must take into account that this test is being used for screening, and a high sensitivity is thus desirable. We therefore propose raising the cut-off point to 0.8 to achieve this high sensitivity and a high negative predictive value, even with a lower specificity. Although there were small differences in the 3 healthcare settings in which the screening studies were performed, it would appear that cut-off points close to 0.8 for the FEV1/FEV6 ratio are those with greatest validity for screening. On this basis, individuals at risk of COPD who have an FEV1/FEV6 ratio greater than or equal to 0.8, as determined by the COPD-6, would have a much lower probability of being diagnosed with COPD or, if this were not detected, they would have mild disease. In these cases, spirometry could theoretically be deferred, and follow-up scheduled. When the result is below 0.8, spirometry should be performed with a bronchodilator test to confirm or exclude the diagnosis of COPD. An AUC value of 0.8 seems also acceptable for screening tests performed in ambulatory settings or by staff not having extensive training in lung function tests.

In the present study we diagnosed COPD in a significant number of at-risk patients: 31.5% in the overall group, and 40% in the PC cohort. The majority of these patients presented a mild or moderate degree of obstruction, though more than 16% of patients had severe obstruction. This would support a recommendation for proactive screening of susceptible individuals in order to initiate appropriate therapeutic strategies at the earliest opportunity[1].

A curious finding in our results was the difference between the percentage of male patients in the CPh cohort (40%) compared with the other 2 cohorts (70.6% and 71.6%). We believe that this simply reflects that women are more likely than men to attend community pharmacies.Several reports have been published on the use of portable devices to screen for COPD in non-specialized settings. Miravitlles et al[17] analysed the diagnostic yield of the FEV1/FEV6 ratio measured using the COPD-6 device to screen for chronic airways obstruction, concluding that it was useful for this purpose and the best cut-off point was 0.75. But the authors did not specify who performed the COPD-6 measurements, a factor that could affect the results. In our study, the measurements in the PC cohort were made by the participating doctors who recruited the patients. This design would provide results that were a more reliable reflection of daily practice, in which all doctors are required to be able to use portable devices as screening tools, even if they have not received extensive specific training on pulmonary function. Frith et al[18] validated another portable device, the PiKo-6, using a spirometry-measured post-bronchodilator FEV1/FVC ratio <0.7 as the gold standard diagnostic criterion. They demonstrated the usefulness of the FEV1/FEV6 ratio for COPD screening in PC (area under the curve 0.85, similar to our results), and proposed a cut-off point of 0.75 for the FEV1/FEV6 ratio (S 81%; Sp71%). In our opinion, if we wish to use these devices for screening, we must look for a high sensitivity, even with a lower specificity. Our cut-off point of 0.8 has also been proposed by other authors[9,19].There are other studies that have observed different AUCs such as the ones performed by Van den Bemt et al[20] or Thorn et al[21]. The reason for the observed differences might be due that they studied populations with different characteristics or to the fact that microspirometry was performed by staff with higher experience in lung function assessment compared with our staff, who received a 2-hour session training.

With regard to other healthcare settings, few studies have been published. In the FarmaEPOC study[22], patients at risk of COPD (selected by a clinical questionnaire) were screened in the pharmacy by conventional spirometry performed by staff trained on a 4-day course. An expert in spirometry telematically reviewed the quality of the studies. Only those patients with an FEV1/FVC <0.7 (without bronchodilatation) were referred to PC to complete the study. The results in PC were only reported to the pharmacies in 15% of cases, which is a significant limitation. Furthermore, it was not explained how the diagnosis of COPD was reached in PC. This screening strategy would require pharmacies to be equipped with spirometers, lengthier training for the staff performing the studies, and time dedication by an expert healthcare professional to review the quality, and it would therefore be much more costly and difficult to apply in the majority of CPh. For that reason, in our CPh cohort, we opted to use a portable device, the COPD-6, much simpler and cheaper than conventional spirometry, an essential aspect if we wish to extend its use widely to CPh. Solidoro et al[23] in Italian pharmacies also used a portable device, the PiKo-6, to detect obstructive and restrictive lung disease, not specifically COPD; furthermore, in contrast to our study, those authors did not take into account whether patients had a previous diagnosis nor did they define the standard comparator test used to confirm the diagnosis of COPD.

There are very few data on the results of COPD screening programmes in ES. Recently, a French group[24] published a study in which they performed COPD screening in an ES, using the Nèo6 device. Spirometry was performed in those who had a questionnaire suggestive of COPD and an FEV1/FEV6 ratio <0.8, the same as the cut-off point that we propose. In the group of patients studied by spirometry, only 8,91% presented COPD, a surprisingly low prevalence of COPD compared with the results from our ES cohort (31.4%).

One of the strengths of our study is that we took the measurements using a baseline COPD-6 test, without bronchodilator, which is how the study would be performed in daily practice in any of the non-specialized settings, and we compared this with spirometry after bronchodilatation, which is the gold standard test for the diagnosis of COPD. This could be one of the reasons for the difference in results compared with our study performed in the hospital setting[12]. A further strength is the sample size, which was over 350 individuals from different settings; this increases the validity of the results obtained. Furthermore, the study was performed on an unselected population that we believe reflects reasonably and accurately the characteristics of the general population. Finally, the fact that the spirometry was performed blinded to the results of the COPD-6 eliminates observer bias and increases the validity of our results. Moreover, our research falls within the scope of COPD research proposed in the official declaration of the American Thoracic Society (ATS) and the European Respiratory Society (ERS)[13], recommending studies that evaluate case-finding strategies using mini-spirometers.

However, there are certain limitations to our study, as the use of a post-bronchodilator FEV1/FVC ratio <0.7 as the gold standard, criticised and questioned for possible inaccuracy[25], but it continues to figure in current guidelines[1,2]. A further limitation could be that the participating healthcare professionals in these studies were highly motivated. We do not know whether our results might be extrapolated to all these settings, although it is likely that with a brief training course on the use of the COPD-6 device, any health professional could use it in daily practice and achieve similar results.

In conclusion, the COPD-6 device is a valid tool for screening for COPD in non-specialized healthcare settings, and can be used by staff who has received a brief training session. For COPD screening, the best cut-off point for the FEV1/FEV6 ratio is 0.8. Obviously, the COPD-6 device will never replace spirometry, but it enables those individuals with COPD high risk to be selected when they visit their PC physician, their local pharmacy or attend an ES for an exacerbation of respiratory symptoms. The proposed cut-off point is highly reliable for excluding those individuals who do not require spirometry due to the very low likelihood that they have the disease. These results are relevant, as they will enable us progressively to reduce COPDunderdiagnosis in a relatively simple and cost-effective manner.
